# Characterising the Quality of Behaviour Driven Development Specifications

**DOI:** 10.1007/978-3-030-49392-9_6

**Published:** 2020-05-06

**Authors:** Leonard Peter Binamungu, Suzanne M. Embury, Nikolaos Konstantinou

**Affiliations:** 6 University of Oslo, Oslo, Norway; 7 Monash University, Clayton, VIC Australia; 8 IT University of Copenhagen, Copenhagen, Denmark; 9 University of British Columbia, Vancouver, BC Canada; Department of Computer Science, The University of Manchester, Oxford Road, Manchester, M13 9PL UK

**Keywords:** Behaviour driven development, Test suite quality, Test suite quality assessment

## Abstract

Behaviour Driven Development (BDD) is an agile testing technique that enables software requirements to be specified as example interactions with the system, using structured natural language. While (in theory) being readable by non-technical stakeholders, the examples can also be executed against the code base to identify behaviours that are not yet correctly implemented. Writing good BDD suites, however, is challenging. A typical suite can contain hundreds of individual scenarios, that must correctly specify the system as a whole as well as individually. Despite much discussion amongst practitioners and in the blogosphere, as yet no formal definition of what makes for a high quality BDD suite has been given. To shed light on this, we surveyed BDD practitioners, asking for their opinions on the quality criteria that are important for BDD suites. We proposed, and asked for opinions on, four quality principles, and gave practitioners the option to add more principles of their own. This paper reports on the results of the survey, and presents an approach to defining BDD suite quality.

## Introduction

Behaviour Driven Development (BDD) [14] enables software requirements to be given as a collection of examples (usually referred to as *scenarios*) that use structured natural language to describe how users will interact with the System Under Test (SUT). A typical BDD suite can contain hundreds of individual scenarios [1], organised as several *feature* files. Listing 1 shows a sample scenario from a feature that specifies customer interactions with an ATM.
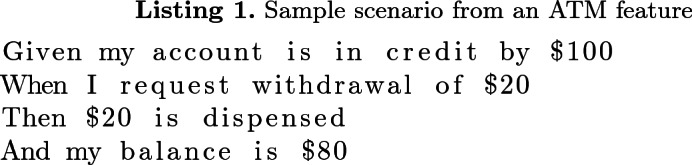


Despite their natural language form, BDD scenarios can be linked to the SUT through *glue code*, allowing them to be executed. This turns the specification into a living document, in which failing scenarios indicate features that are not yet fully or correctly implemented. The following is an example of Java glue code for the second step in the scenario in Listing 1:
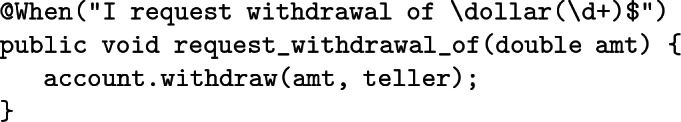


The annotation for the method contains a regular expression that is matched against each scenario step as it is executed. When a method is found with a matching annotation, it is executed, with the values extracted from the capture groups passed as the parameter values. Literature has reported both the benefits of using BDD and the challenges that software teams face when using BDD (e.g. [1, 13]).

BDD approaches fit well with other agile practices for requirements gathering and documentation, with BDD features mapping naturally to user stories and the individual scenarios mapping (though more loosely) to the conditions of satisfaction sometimes documented on user story cards as the confirmation element of the story. The fact that BDD scenarios are expressed using customer languages means that they can (in theory, at least) be read and understood by non-technical project stakeholders, and compared with their knowledge of the domain. BDD is thus typically characterised as a customer-facing form of testing, that can be undertaken from the earliest stages of the project, once the first user stories have been identified, and that delivers value right through development and (in a regression testing role) the operational lifetime of the software.

Writing a high quality BDD suite is important. BDD suites can quickly grow to include hundreds or even thousands of individual scenarios [1]. The suite must specify the correct behaviour as a whole, as well as through the individual scenarios. For the long term correctness and extensibility of the system, it is important that the BDD suite be written to a high standard. BDD suite quality has been heavily discussed amongst practitioners and in the blogosphere, and is beginning to be considered by the software engineering research community [15–17] but no formal notion of BDD suite quality has been given that can assess individual scenarios and their relation to the rest of the suite.

In this paper, we present the results of a survey of BDD practitioners’ views about BDD suite quality. To give structure and precision to the results, we proposed four principles of BDD suite quality, and asked respondents to give their level of agreement with them. We also asked respondents to describe additional quality principles that they thought were important, and that weren’t covered by the proposed principles. All the four principles received support, with at least 75% of respondents voting in support of each one, though all of them also received a number of dissenting votes. Respondents also stressed the importance of writing scenarios in way that promotes reuse within BDD, but put most emphasis on readability and clarity of the resulting specification.

This paper makes three contributions:

1. **BDD Suite Quality Principles:** We propose four principles describing features expected of a high quality BDD specification.
2. **Practitioner Support for the BDD Suite Quality Principles:** We report the results of a survey of practitioner support for the BDD suite quality principles.
3. **Other BDD Suite Quality Aspects:** We report about other quality aspects of BDD suites, from which further quality principles can be developed.

The rest of the paper is structured as follows: Sect. 2 surveys related work on test suite quality; Sect. 3 presents the approach we used to obtain quality principles, and the quality principles themselves; Sect. 4 presents practitioners’ opinions about the proposed principles; and Sect. 5 concludes the paper and highlights future research directions.

## Related Work

In this section, we first explore how quality is characterised in automated test suites more generally, to see whether these notions of quality can inform the definition of quality for BDD. After that, we review the literature on BDD quality specifically.

**Assessing the quality of tests and requirements:** Tengeri *et al.* [21] devised a method for test suite improvement based on test coverage proportions. To use the method, an improvement goal is first set (e.g removing duplicate test cases, improving coverage of some parts of code, etc.). Then a granularity of focus is chosen–coarse (e.g. functional level) or fine (e.g. statement level). Various metrics are then computed based on coverage data gathered during test execution, which are then used to inform the process of updating tests and code.

Palomba *et al.* [18] found that test cohesion and test coupling are important criteria for increasing the quality of automatically generated test cases, and included these criteria in their algorithm for search-based test case generation. Meszaros [12] defines test cohesion and coupling as follows. Test cohesion refers to the simplicity of a test case–a highly cohesive test case should not be involved in the verification of a lot of functionality. Test coupling, on the other hand, measures the extent to which tests overlap with each other. To be easily maintainable, tests should have low cohesion and coupling. Improvement in quality of automatically generated test cases was observed when the two criteria were incorporated into an algorithm for automatic test case generation [7].

Daka *et al.* [5] used human judgement to develop a model for assessing the readability of unit tests, and then applied this model to generate readable unit tests. Crowdsourcing was used to rate the readability of tests on a five point scale. After that, 24 structural, complexity, and code density unit test features were selected and used to build the model. When compared with the crowd-sourced readability results, the model was found to be in agreement by 89%. Moreover, using the model to augment automatic generation of unit tests, it was found that more readable unit tests were generated, and the speed at which humans could answer questions about maintenance increased by 14% without losing accuracy.

There have been a small number of attempts to assess the quality of natural language tests and requirements through the notion of *smells*. Examples are the work of Hauptmann *et al.* [9] in which a set of smells in manual natural language tests was proposed, along with ways to detect them, and the work of Femmer *et al.* [6] in which nine smells in natural language requirements (and methods for their detection) were proposed.

**Assessing the quality of BDD suites:** Cochran *et al.* proposed a tool to detect smells in BDD suites [4]. Their work is similar to ours in a sense that it is also about the quality of BDD feature suites. However, the tool does not provide a mechanism to assess the quality of a scenario with respect to all other scenarios in a feature suite.

To the best of our knowledge, the work of Oliveira *et al.* [15–17] is the only published work that focuses on quality in BDD specifications. Specifically, Oliveira *et al.* suggested that a good BDD scenario should be *essential*, *focused*, *singular*, *clear*, *complete*, *unique*, *ubiquitous*, and *integrous* [15–17]. However, these attributes define, in general terms, the characteristics expected of a good scenario, but are not precise enough to facilitate the assessment of the quality of one scenario in relation to all other scenarios in a suite.

## BDD Suite Quality Principles

In this section, we first present the process used to produce the principles, and then we describe the four principles in their general form.

### Aspects of Quality in BDD Specifications

To understand what constitutes good quality in BDD suites, we first searched the scientific literature for attempts to define quality in BDD specifications. This gave us only the work of Oliveira *et al.* which suggested that good BDD scenarios should be *essential*, *focused*, *singular*, *clear*, *complete*, *unique*, *ubiquitous*, and *integrous* [15–17]. However, these attributes define, in more general terms, the characteristics expected of a good scenario, but are not precise enough to facilitate the assessment of the quality of one scenario in relation to all other scenarios in a suite. Thus, BDD quality facets in the literature have focused on quality at the scenario level, when the present work is interested in quality at the suite level.

To obtain attributes that are suitable for assessing the quality of a scenario relative to all other scenarios across a feature suite, we borrowed ideas from the quality attributes in the work of Oliveira *et al.* [17] and complemented these ideas with other practitioners’ opinions on quality in BDD feature suites. To obtain practitioners’ opinions on quality in BDD feature suites, we analysed articles from the BDD Addict Newsletter [20], a monthly online newsletter about BDD, which publishes articles about various aspects of BDD from the perspective of BDD practitioners. Articles from 32 issues of the newsletter (from February 2016, when the first issue was released, to December 2018) were analysed for quality facets in BDD suites. We then searched StackOverflow^1^ and StackExchange^2^ for any additional BDD quality facets that might not have been covered in the BDD Addict Newsletter.

Table 1 summarises the quality facets we obtained from both scientific and grey literature. Some of these quality facets focus on the step level, others focus on the scenario level, and still others focus on the suite level.

**Table 1.** BDD quality aspects from scientific and grey literature

**S/n**	**Quality Aspect**
1	A good quality scenario should be concise, testable, understandable, unambiguous, complete, and valuable
2	Reuse of steps across scenarios can improve suite quality
3	Declarative (high level) steps are preferred to imperative (low level) steps
4	Business terminology should be consistently used across the specification
5	Scenarios should focus on the benefit they offer to users, if implemented
6	Scenarios should use the terminology understood by all project stakeholders
7	Each scenario should test one thing
8	Scenario titles should be clear
9	Scenario descriptions should be focused
10	Personal pronoun “I” should be avoided in steps
11	Too obvious and obsolete scenarios should be avoided in the suite
12	Scenario outlines should be used sparingly
13	Scenarios should clearly separate Given, When and Then steps
14	Use past tense for contexts (Given), present tense for events (When), and “should” for outcomes (Then)

The review of both scientific and grey literature resulted in a useful set of quality notions for general use, but none of them were sufficiently precise to allow, for example, a tool to be created to find violations or propose improvements. We selected 4 of these notions for further analysis, based on their potential to be precisely defined, and created from them four hypothesised principles to be tested against community opinion. These principles are presented in the next four subsections.

### Principle of Conservation of Steps

A BDD scenario consists of a sequence of steps, as illustrated in the simple banking example in Listing 1. The Conservation of Steps principle seeks to maximise the use of existing step phrases across the suite, and tries to avoid having too many step phrases that are used only in one or two scenarios. To illustrate this idea, suppose we need to write a scenario for when the bank customer tries to withdraw more money than is in their account; this principle suggests we should reuse the step phrases from the existing scenarios rather than inventing new ways of phrasing the same idea (e.g. “my account is in credit by $10” rather than “my account balance is $10”).

This principle is based on the rationale that the steps in a BDD suite form a vocabulary for talking about the functionality of the system. The Given and Then steps describe different aspects of the system state, while the When steps describe all the state-changing actions the completed system should be able to take. If the same functionality can be expressed using a smaller number of steps, that should reduce the comprehension effort needed to understand the whole suite, as well as reducing the chance that duplicated or subtly inconsistent scenarios will be added in future.

### Principle of Conservation of Domain Vocabulary

Any organisational process that is supported by software will typically accrue over its lifetime a set of terms or phrases describing the key ideas in the domain of the process that are used by the people involved to communicate about and advance the state of the work. The *Ubiquitous Language* agile practice requires the software team to use the same terms wherever possible, in the artefacts that describe and constitute the system [19]. This is also true within BDD suites. For example, in the scenario in Listing 1, it would be desirable to use the term “balance” whenever referring to the amount funds remaining in an account, instead of inventing new phrases which might be synonymous to “balance”.

With this in mind, the Principle of Conservation of Domain Vocabulary seeks to maximise the value of each domain term or phrase that is used in the BDD suite. Inevitably, in any human endeavour, duplicate terms may be used for the same concept. But each additional term increases the cognitive load for readers and writers of scenarios. We therefore consider a suite to be of high quality if it can express the required semantics clearly with the minimum number of domain terms and phrases.

### Principle of Elimination of Technical Vocabulary

Since BDD scenarios in a suite are meant to be readable by all project stakeholders (including end users), the use of technical terms that, in most cases, only the development team can understand, is discouraged. For instance, in the “When” step of the scenario in Listing 1, use of the phrase “I click the button for withdrawing $20” would reduce the chances of comprehension by some end users, as well as imposing design choices onto the specification that may be non-optimal in the implemented system. As such, scenarios that use domain terms are generally preferred to scenarios that use technical terms. This principle, therefore, focuses on minimising the use of technical terms in the steps of BDD scenarios across the suite.

### Principle of Conservation of Proper Abstraction

One challenging aspect in the creation of a BDD feature suite is to select an appropriate level of abstraction for the scenarios, and in particular for the steps. Higher level steps convey more semantics, so that scenarios can be expressed using fewer steps, and are often closer to the domain concepts that end users are familiar with. But they require more extensive glue code to be written, with more embedded assumptions, so that sometimes the meaning of the suite cannot be understood with precision without reference to the glue code. Lower level steps describe more fine-grained aspects of system state and state change. Scenarios using them will typically be longer, requiring more steps to express the same semantics than when using higher level steps. But lower level steps require smaller simpler glue code to implement them. Feature suites written using very low level steps can be too procedural, resembling traditional testing scripts, rather than end-user focused declarative examples.

In Listing 2 which shows a scenario that belongs to the same feature as that in Listing 1, the “Given” condition (which could be expressed as “my account is in credit by $10”) is broken into two lower level steps on lines 2 and 3. This introduces some inconsistency in the abstraction levels of steps in the two scenarios (the one in Listing 1 and the one in Listing 2), though both scenarios belong to the same feature. Such inconsistency could also manifest in scenarios across different features of the same BDD suite.
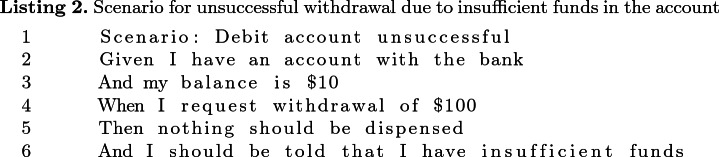


Intuitively, therefore, a BDD feature suite in which scenarios are written at a consistent level of abstraction will be easier to understand, extend and maintain. On the contrary, if the feature suite has a mix of scenarios expressed at a low level of abstraction and scenarios expressed at a higher level of abstraction, it can be difficult for a maintenance engineer to decide on the level of abstraction to use in expressing a new scenario. Moreover, there is likely to be duplication of steps and glue code, and the test harness code will also be at inconsistent levels of abstraction, adding to the comprehension and maintenance burden.

## Community Support for the BDD Quality Principles

We used a survey with 9 questions to gather practitioners’ opinions on the 4 principles. We wanted to discover whether the principles resonated with practitioners as meaningful facets of BDD suite quality, and to discover whether there were important quality facets we had overlooked.

### Survey Design

The survey questions covered respondents’ demographics, views on the four principles and opinions on quality aspects not covered by them. The questions on demographics were:

- *Q1: Which of the following best describes your job?*
- *Q2: What is the size of your organisation?*
- *Q3: Which of the following best describes your experience of working with BDD?*
- *Q4: What country are you based in?*

To mitigate the potential for bias and allow respondents to react in a natural way, the principles were not formally disclosed in the survey. Instead, we sought respondents’ degree of agreement with informal statements of the principles. Thus, the next four questions, Q5 through Q8, are respectively informal statements for Conservation of Steps, Conservation of Domain Vocabulary, Elimination of Technical Vocabulary, and Conservation of Proper Abstraction.

- *Q5: When adding new scenarios to a BDD suite, we should strive to reuse existing steps wherever that is possible without compromising readability of the scenario.*
- *Q6: When writing the BDD scenarios for a particular domain, we should strive to make use of a minimal set of domain terms in our scenario steps. That is, we prefer to write steps that reuse domain terms already used in other steps, rather than introducing new terms, wherever that is possible without compromising readability of the scenario.*
- *Q7: When adding new scenarios to a feature suite, we should prefer to use steps that are expressed using domain terms over steps that are expressed using more technical language, whenever we have a choice.*
- *Q8: Within a feature suite, the abstraction levels of steps in one scenario should be largely consistent with the abstraction levels of steps in other scenarios in the suite.*

We then added a question for respondents to mention any other BDD suite quality facets that might not have been captured by the four principles:

- *Q9: Please give us any other thoughts on how to keep scenarios and steps of a BDD specification readable, easy to extend, and maintainable.*

Questions 1 to 3 presented respondents with options to choose from, while question 4 was free text. Questions 5–8 asked respondents to indicate their degree of agreement, on a Likert scale, with each of the given statements. An “other” free text option allowed respondents to provide alternative responses or to qualify their degree of agreement. Question 8 was supplemented by 2 example scenarios, clarifying the meaning of “abstraction level”. Question 9 allowed free text for respondents to freely describe additional quality aspects.

The survey was pretested on a BDD practitioner. It was deployed using SelectSurvey.NET on our university’s servers, and ran for one month from December 2018.

### Respondents and Their Demographics

We distributed the survey through a convenience sample using online discussion groups and personal emails. Although this approach to sampling limits the ability to generalise from the findings, convenience sampling is the recommended pragmatic alternative when probabilistic sampling is not possible [8]. The survey was posted to several Google Groups^3^ and through an e-mail list of 500+ contributors to BDD projects in GitHub^4^, supplemented by our personal industry contacts. Since we requested respondents to share the survey with other interested parties, some respondents might have been recruited through snowballing. Kochhar *et al.* [10] and Cito *et al.* [3] used similar methods to recruit survey respondents.

The survey was viewed by 129 people, of whom 56 submitted responses to the questions on BDD suite quality. Hereafter, all discussions of survey results refer to this subset of 56 respondents. We randomly assigned numbers to each respondent and refer to them as R1 to R56. The number of responses to questions on the four principles were: Conservation of Steps (55), Conservation of Domain Vocabulary (54), Elimination of Technical Vocabulary (55), and Conservation of Proper Abstraction (56). Question 9, which asked about quality aspects not covered by the principles, received 31 responses.

The distribution of respondent roles was: Developer (60.7%), Tester (12.5%), Consultant (7.1%), Chief Technology Officer (CTO) (5.4%), Researcher (3.6%), Business Analyst (1.8%), Other (7.1%), and did not say (1.8%). The sizes of respondent organisations were: 1–20 employees (26.8%), 21–99 employees (16.1%), 100–1000 employees (26.8%), more than 1000 employees (21.4%), all sizes (7.1%), did not mention (1.8%). Respondents’ experience of working with BDD were: 1 year (7.1%), 1–5 years (28.6%), 6–10 years (51.8%), and 10 years (12.5%). Finally, the geographical distribution of respondents were: Europe (64.3%), North America (21.4%), Asia (5.4%), Zealandia (7.1%), and did not say (1.8%).

### Survey Data Analysis

We first plotted the respondents’ levels of agreement for each principle, and summarised other respondents’ comments on each principle. Then, we used the thematic analysis guidelines by Braun and Clarke [2] to analyse the free text responses on other ways to keep BDD suites comprehensible, extensible and maintainable. In particular, we conducted *theoretical thematic analysis* [11], in which data analysis is guided by the research question; in our case, the question of interest was how to keep BDD specifications readable, extensible and maintainable.

**Fig. 1.**
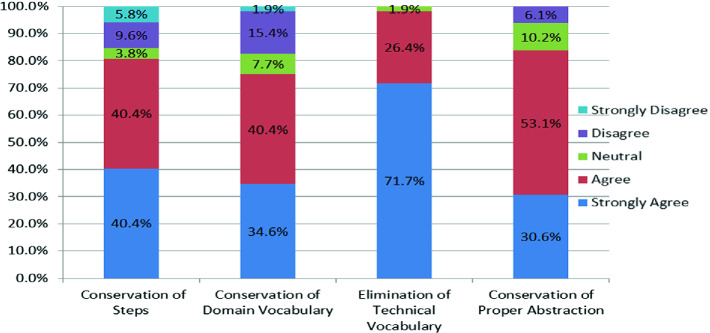
Acceptability of each BDD quality principle by respondents

Data coding began after an initial pass through the responses. We coded everything in the text that related to readability, extensibility and comprehensibility of BDD specifications. We used open coding—we had no predetermined codes. After coding, we grouped related codes together to form the list of initial themes which we iteratively refined to produce the final list. Finally, we categorised the themes as actionable points either for all project stakeholders or for developers/QAs.

### Survey Results

Figure 1 shows the respondents’ degree of agreement with each principle. Each principle was accepted (strongly agree/agree) by at least 75% of the respondents who answered the question and clearly indicated their degree of agreement.

Other comments on the principles were as follows.

- **Conservation of Steps:** steps should also be expressed in general terms; sometimes it can be a good idea to focus on writing clear steps that serve the purpose, and then fix the design later; the main focus should be on the readability, and reuse of steps can affect the readability and maintainability of the specification.
- **Conservation of Domain Vocabulary:** it should be possible to use new domain terms whenever necessary, provided that the specification remains readable to customers.
- **Elimination of Technical Vocabulary:** implementation words can sometimes be used, depending on the product owner and expected readers of the specification; sometimes, it can be challenging to translate domain words used in scenarios into implementation details.
- **Conservation of Proper Abstraction:** the abstraction levels should be determined by capturing correct requirements, and producing scenarios that are readable to customers; lower abstraction levels can be appropriate if scenarios carry data; sometimes, one can use different abstraction levels for Given, When, and Then steps.

Responses to question 9, requesting other opinions on facets of BDD quality, are summarised in Table 2 and Table 3.

**Table 2.** Other recommended practices for all project stakeholders on how to keep BDD specifications readable, easy to extend, and maintainable

**S/n**	**Theme**	**Frequency**	**Sample Excerpts**
1	Specification should act as readable business documentation	11	-*“The key is to have a multi-layered approach; the gherkin scenarios should focus on being readable as business documentation...”* (R8, Consultant)
2	Clear description of business goals using examples	5	-*“Describe the business goal and the steps on how to achieve them as clearly as you understand at the moment.”* (R4, Developer) -*“Focus on clean specifications that are consistent within the bounded context”* (R6, IT Consultant)
3	Use of common domain concepts and terms across the specification	5	- *“...I like the idea of a glossary of terms from the Writing Great Specifications book...”* (R6, IT Consultant) -*“Use the same domain language and terminology as the rest of your organisation/customers/industry”* (R26, Chief Technology Officer) -*“...Have a glossary with important domain concepts, primary term and possible synonyms.”* (R44, Principal Software Architect)
4	Focus on capturing comprehensive requirements for all project stakeholders	5	- *“BDD specification should satisfy both business analyst and developer as much as possible.”* (R11, Developer) - *“Everything around BDD and Specification by example is around creating a shared understanding. That is the core reason to do examples in the first place; the help us uncover hidden assumptions...”* (R18, BDD Coach)
5	Specification should be easy to understand based on general domain knowledge	4	-*“Test them on other people not involved in the project. Can they understand what they mean? Can they determine the intent of each scenario?... ”* (R2, Consultant) -*“Where possible, involve less technical stakeholders and team members in the process of scenario development...”* (R46, Developer)
6	Share specs with stakeholders for reference and correction, and perform regular maintenance of specs	4	-*“I believe the key would be to periodically revisit them and keep updated, if necessary rewrite or reword older ones. I find it very useful to also publish scenarios using ci tools somewhere so business people can read specs and spot inconsistencies”* (R47, Developer) -*“At the very least, have the specs available for reference by the project stakeholders.”* (R46, Developer) -*“...Refactoring also applies to BDD scenarios...”* (R44, Principal Software Architect)

**Table 3.** Other recommended practices for QAs and developers on how to keep BDD specifications readable, easy to extend, and maintainable

**S/n**	**Theme**	**Frequency**	**Sample Excerpts**
1	Write reusable and yet focused steps and step definitions	11	-*“...the gherkin scenarios should focus on being readable as business documentation, and map to reusable steps in the step definitions. It is the DSL code in the step definitions where the real reusability benefits occur”* (R8, Consultant) -*“It’s best to re-use steps either by referring to them directly (Using Given, And...), or creating a new step definition using the underlying API, not calling one step definition from another”* (R24, Software Engineer in Test)
2	Aim for more stateless scenarios	4	-*“The scenarios should be stateless, in the sense that they should store as few data as possible.”* (R50, Developer)
3	Proper use and order of Given, When, and Then steps; and careful choice and use of framework-specific BDD features	4	-*“Ensure that WHEN’s only perform actions and THEM’s only assert ( do not modify the SUT state ) and are expressed as such”* (R43, Tester) -*“...Choose good titles (scenario/feature) 9) Don’t send test data from feature file, (but examples of behavior are allowed)10) Less is More 11) Limit one feature per feature file. This makes it easy to find features. 12) Hide data in the automation 13) Steps order must be given/when/then - not other order‘”* (R25, Test Architect)
4	Miscellaneous: Keeping an inventory of all steps in a project; clear separation of customer-readable tests from glue code and the underlying API; and leveraging the full capabilities of underlying BDD framework and regular expressions	3	-*“I’m not aware if this is already possible but it would be helpful to produce a dictionary of all the steps used in a project by extracting them from the feature suites.”* (R1, Developer) -*“The key is to have a multi-layered approach; the gherkin scenarios should focus on being readable as business documentation, and map to reusable steps in the step definitions. It is the DSL code in the step definitions where the real reusability benefits occur”* (R8, Consultant) -*“Make full use of the underlying BDD framework / regular expressions and craft the step definitions like a powerful text-based API.”* (R33, Developer)

### Discussion and Threats to Validity

In general, the majority of the respondents supported the principles as acceptable descriptors of facets of BDD suite quality (see “Strongly Agree” and “Agree” responses in Fig. 1). The written comments stressed the importance of reuse within BDD, but put most emphasis on readability and clarity of the resulting specifications. For all the principles, the respondents who stated the reasons for dissenting mainly emphasised that decisions on reuse or use of steps, domain terms, implementation terms and abstraction levels should be determined by the specific contexts.

Moreover, even the other quality facets mentioned by the respondents (Table 2 and Table 3) resonate well with the quality principles we have proposed. For example, themes 3 and 5 in Table 2 respectively resonate with the Principle of Conservation of Domain Vocabulary and the Principle of Elimination of Technical Vocabulary; at the same time, theme 4 in Table 3 proposes keeping an inventory of all steps in a suite, which would be a facilitative environment for the implementation of the Principle of Conservation of Steps.

The threats to the validity of our results are the following:

- We mainly depended on practitioners with online presence, either through GitHub or other online forums where BDD and other agile topics are discussed. Thus, we might have missed some in-house practitioners that are not easily reachable through any of those means. To mitigate the effects of this, we requested those who completed or saw the survey to refer it to others. Also, we sent survey completion requests to some practitioners who were known in person to the authors, and requested them to share the survey to others.
- The four quality principles we propose were partly influenced by our choices of what to focus on in order to come up with an initial set of BDD suite quality principles for testing against community opinion (Sect. 3.1). To mitigate the effects of this, our choices of quality aspects to focus on were mainly informed by the quality facets from the state-of-the-art and the state-of-practice (Sect. 3.1). Moreover, all the principles were supported by majority of survey respondents from the BDD practitioner community (Fig. 1).
- Most of the respondents might have been using a particular BDD tool, so that our results could be valid for users of a specific BDD tool only. To cover practitioners using a variety of BDD tools, we followed the objective criteria mentioned in Sect. 4.2 to identify email addresses to which survey completion requests were sent. We also posted the survey in a general BDD forum, in anticipation that respondents from that forum might be using different tools.
- The use of convenience sampling (in our case, depending on self-selecting respondents within the groups we contacted) might limit the ability to generalise from the survey findings. To mitigate the effects of this, we survey 56 respondents from 5 continents across the world (Sect. 4.2), and some of the respondents were contributors to sizeable BDD projects in GitHub (Sect. 4.2). Still, our results may not generalise to all BDD practitioners across the world. For example, our results do not represent BDD practitioners who are not proficient in English.

## Conclusions

BDD is currently used by industry teams to specify software requirements in a customer understandable language [1]. This produces a collection of examples that act as executable tests for checking the behaviour of the SUT against the specifications. However, large volumes of BDD suites can be hard to understand, maintain and extend. Duplication, for example, can be introduced by members joining the teams at different points in time.

We have proposed four principles for assessing the quality of BDD suites. Each principle was supported by at least 75% of the practitioners we surveyed. Practitioners also emphasised the importance of reuse within BDD, but stressed more on readability and clarity of the resulting specifications.

In the future, we will investigate the operationalisation of these principles, so that they can be used to assess the quality of BDD suites. Moreover, we will investigate the possibility of developing and evaluating more principles from other issues reported by practitioners (Table 1, Table 2, and Table 3). For example, we need a principle on “readability” of scenarios in a suite, a property that was rated highly by the survey respondents, and that probably trumps all over other quality principles. Respondents would prefer to keep the scenario that breaks our rules if it is the more readable one. This suggests a future work idea, looking for general metrics of text readability, to see if they can be applied to BDD suites. As well, we will investigate novel ways to help practitioners to manage steps, terms and abstraction levels of specifications. It might also be worthwhile investigating how the quality of BDD feature suites is related to the overall system quality, to inform software quality planning in organisations.

## Footnotes

1 https://stackoverflow.com/.

2 https://stackexchange.com/.

3 Cucumber, Behaviour Driven Development, Specflow, Concordion, and Serenity BDD.

4 The list was harvested through a process described elsewhere [1].
